# The Effect of Different Extraction Protocols on *Brassica olerace*a var. *acephala* Antioxidant Activity, Bioactive Compounds, and Sugar Profile

**DOI:** 10.3390/plants9121792

**Published:** 2020-12-17

**Authors:** Nikola Major, Bernard Prekalj, Josipa Perković, Dean Ban, Zoran Užila, Smiljana Goreta Ban

**Affiliations:** 1 Institute of Agriculture and Tourism, K. Huguesa 8, 52440 Poreč, Croatia; bernard@iptpo.hr (B.P.); josipa@iptpo.hr (J.P.); dean@iptpo.hr (D.B.); zoran@iptpo.hr (Z.U.); 2 Centre of Excellence for Biodiversity and Molecular Plant Breeding, Svetošimunska 25, 10000 Zagreb, Croatia

**Keywords:** antioxidant activity, extraction, glucosinolates, kale (*Brassica oleracea* var. *acephala*), phenolics

## Abstract

The extraction of glucosinolates in boiling aqueous methanol from freeze dried leaf tissues is the most common method for myrosinase inactivation but can be hazardous because of methanol toxicity. Although freeze drying is the best dehydration method in terms of nutritional quality preservation, the main drawbacks are a limited sample quantity that can be processed simultaneously, a long processing time, and high energy consumption. Therefore, the aim of this study is to evaluate the effects of applying high temperature for myrosinase inactivation via hot air drying prior to the extraction step, as well as the effects of cold aqueous methanol extraction on total antioxidant activity, total glucosinolates, total phenolic content, and sugar profile in 36 landraces of kale. The results from our study indicate that cold aqueous methanol can be used instead of boiling aqueous methanol with no adverse effects on total glucosinolate content. Our results also show that hot air drying, compared to freeze drying, followed by cold extraction has an adverse effect on antioxidant activity measured by DPPH radical scavenging, total glucosinolate content, as well as on the content of all investigated sugars.

## 1. Introduction

Vegetables from the Brassicaceae family are known for their excellent nutritional value and are abundant in carbohydrates, vitamins (ascorbic acid, folic acid, β-carotene, α-tocopherol), macro and micro elements (iron, calcium, selenium, copper, manganese, zinc), as well as secondary metabolites, including glucosinolates, phenolics (tannins, phenolic acids, anthocyanidins, flavonols, coumarins, flavones), and other bioactive molecules, such as phytosterols and terpenoids [1,2]. They are also known for their antimicrobial and anticancerogenic activity [3,4]. One of the well-known members of the Brassicaceae family is kale (*Brassica oleracea* var. *acephala*). It originates and is traditionally used in Mediterranean countries, but has gained special attention and popularity in the U.S., and later worldwide over the last decade [5]. It is characterized by leaves, which do not form a head, unlike other leafy vegetables from the Brassicaceae family as white cabbage, savoy cabbage, Brussels sprouts, and Chinese cabbage [6].

The most abundant sulfur containing compounds in plants from the Brassicaceae family are glucosinolates and S-methylcysteine sulfoxide [7]. Cruciferous vegetables are extensively researched regarding their beneficial compounds especially ones active in cancer prevention. Isothiocyanates are degradation products from glucosinolates are known for their antioxidant, immunostimulatory, anti-inflammatory, antiviral and antibacterial properties [8,9,10]. Isothiocyanates are converted from glucosinolates in a reaction catalyzed by the enzyme myrosinase which activates and releases upon plant tissue injury. The myrosinase enzyme was first discovered in 1840 from the mustard seed after which the Brassica studies were focused on clinical studies and prospective benefits of cruciferous vegetables [1]. Myrosinase catalyzes glucosinolate hydrolysis after the plant tissue is injured or in any way disrupted. Therefore, accurate glucosinolate analysis depends on myrosinase inactivation [11]. Differences in glucosinolate content regarding Brassica species and environmental factors can be an obstacle when comparing different studies regarding nutritional and bioactive quality of cruciferous vegetables. Moreover, presence of the enzyme myrosinase, activated in plant handling and extraction processes that precede glucosinolate determinations, is also an aggravating circumstance, which can lead to a decrease in total glucosinolate concentration [12]. Most published glucosinolate analysis methods employ dehydration by freeze drying for myrosinase inactivation and denaturation by moderately high temperature during the glucosinolate extraction step [12,13]. This process is outlined in the ISO9167:2019 standard, as well as in the work by Grosser and van Dame [14]. Freeze drying is a dehydration process based on water sublimation under vacuum, which protects the primary structure and shape of the sample [15]. Although freeze drying is considered the best form of dehydration, the process is time consuming and relatively expensive [16], especially for high throughput methods. On the other hand, hot air drying is simple, fast, and can handle large quantities of samples. 

Most published methods for glucosinolate analysis employ boiling aqueous methanol (70/30, methanol/water, *v*/*v*) in the glucosinolate extraction step [17,18,19,20,21]. Methanol is a common organic solvent and reagent in organic synthetic procedures [22]. Poisoning can occur via methanol ingestion, skin absorption, or inhalation [23]. Acute methanol toxicity evolves in a well-understood pattern and results in metabolic acidosis via formic acid formation and superimposed toxicity to the visual system [24]. The use of boiling methanol in the glucosinolates extraction step increases the risk of poisoning and researchers offered several less hazardous alternatives to the standard method, including the use of hot water or the use of cold (ambient temperature) aqueous methanol [12]. The 2019 revision of the ISO9167 method for the determination of glucosinolates in rapeseed and rapeseed meals by HPLC replaces aqueous methanol (70/30, *v*/*v*) by aqueous ethanol (50/50, *v*/*v*) for lower toxicity [25]. Although the ISO9167:2019 official method supports the use of ethanol instead of methanol for glucosinolate extraction in rapeseed and rapeseed meals, aqueous methanol is the recommended extraction solvent due to the structural and biological diversity of seed and plant matrices, but can be replaced by other solvents if appropriate validation procedures are applied [26]. 

The effect of hot air drying versus freeze drying on phenolic compounds and antioxidant activity was a subject of numerous studies [27,28,29,30] but there is not enough data on the possible effects on glucosinolate and sugar content. While there are several studies concerning the comparison of cold versus hot methanol extraction [12] on glucosinolate levels, the number of samples involved could have been higher, and the studies did not include other bioactive compounds or antioxidant activity in the comparison. 

Therefore, the aim of this work is to evaluate the effect of applying high temperature for myrosinase inactivation via hot air (oven) drying prior to the extraction step, as well as the effect of cold (ambient temperature) aqueous methanol extraction on total glucosinolates content, total phenolic content, total antioxidant activity, and sugar content in 36 landraces of kale (*Brassica oleracea* var. *acephala*).

## 2. Results

### 2.1. The Effect of Different Extraction Protocols on Antioxidant Activity, Bioactive Compounds, and Sugar Content in Brassica

**Figure 1:**
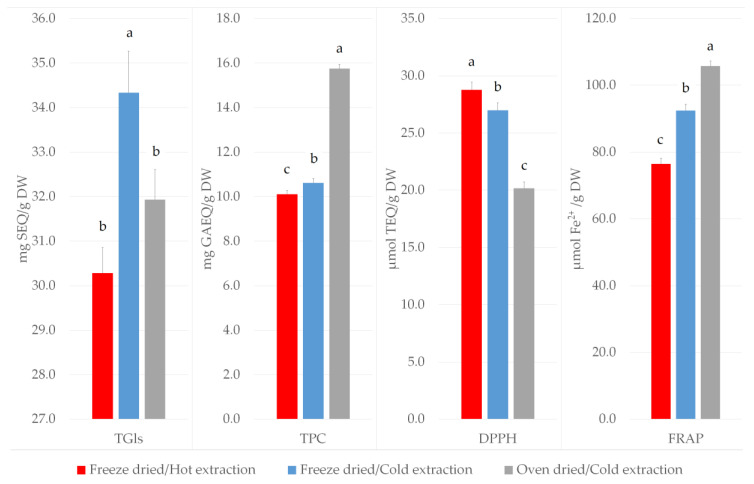
The effect of different extraction protocols on total glucosinolates content, total phenolic content, DPPH radical scavenging activity and ferric ion reducing antioxidant power in *B. oleracea* var. acephala leaf extracts. Values are expressed as mean ± SE (N = 324). The different letters above bars denote significant difference by Tukey’s Unequal N Honestly Significant Difference (HSD) test, *p* < 0.05. TGls—total glucosinolates; TPC—total phenolic content; DPPH—DPPH radical scavenging activity; FRAP—Ferric ion Reducing Antioxidant Power.

The extraction of glucosinolates by the hot methanol extraction step from freeze dried kale leaves yielded significantly lower amounts of total glucosinolates (30.3 ± 0.6 mg sinigrin equivalents (SEQ)/g dry weight (DW), compared to the cold methanol extraction step (34.3 ± 0.9 mg SEQ/g DW) (Figure 1). Significantly higher content of total glucosinolates was found in extracts obtained from freeze dried kale leaves (34.3 ± 0.9 mg SEQ/g DW) compared to oven dried leaves followed by cold extraction (31.9 ± 0.7 mg SEQ/g DW) (Figure 1). 

The same effect was observed for total phenolic content, where significantly lower amounts were extracted with the hot methanol method (10.1 ± 0.2 mg gallic acid equivalents (GAEQ)/g DW) compared to the cold methanol extraction (10.6 ± 0.2 mg GAEQ/g DW) in freeze dried leaves (Figure 1). On the other hand, total phenolic content was found to be higher in oven dried leaves (15.7 ± 0.2 mg GAEQ/g DW) compared to freeze dried B. oleracea var. acephala leaves followed by cold extraction (10.6 ± 0.2 mg GAEQ/g DW) (Figure 1).

As for the antioxidant activity we found significantly higher Ferric Reducing Antioxidant Power (FRAP) values from samples extracted from freeze dried leaves with cold methanol (92.5 ± 1.8 µmol Fe^2+^/g DW) compared to hot methanol extraction (76.5 ± 1.7 µmol Fe^2+^/g DW) (Figure 1). The hot methanol extraction method yielded higher antioxidant power measured by 2,2-diphenyl-1-picrylhydrazyl (DPPH) radical scavenging activity (28.8 ± 0.7 µmol Trolox equivalents (TEQ)/g DW) compared to the cold methanol extraction method (27.0 ± 0.7 µmol TEQ/g DW) from freeze dried B. oleracea var. acephala leaves (Figure 1). DPPH radical scavenging was found to be significantly higher in freeze dried B. oleracea var. acephala leaves (27.0 ± 0.7 µmol TEQ/g DW) compared to oven dried samples (20.2 ± 0.6 µmol TEQ/g DW) followed by cold extraction (Figure 1). FRAP values were significantly higher in oven dried B. oleracea var. acephala leaves (105.7 ± 1.5 µmol Fe^2+^/g DW) compared to freeze dried leaves (92.5 ± 1.8 µmol Fe^2+^/g DW) followed by cold extraction (Figure 1). 

**Figure 2:**
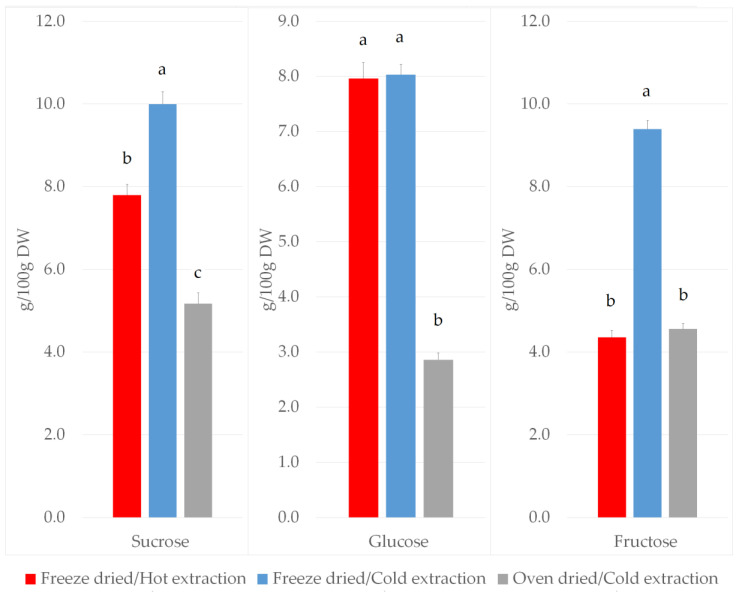
The effect of different extraction protocols on sucrose, glucose and fructose content in *B. oleracea* var. acephala extracts. Values are expressed as mean ± SE (N = 324). The different letters above bars denote significant difference by Tukey’s Unequal N HSD test, *p* < 0.05.

Both sucrose and fructose content was significantly lower in the freeze dried extracts obtained by the hot methanol extraction method (7.8 ± 0.3 g/100g DW and 4.4 ± 0.2 g/100g DW for sucrose and fructose, respectively) compared to the cold methanol extraction method (10.0 ± 0.3 g/100g DW and 9.4 ± 0.2 g/100g DW for sucrose and fructose, respectively) (Figure 2). Glucose content was found to be no different in freeze dried sample extracts obtained by hot or cold methanol extraction (8.0 ± 0.3 g/100g DW and 8.0 ± 0.2 g/100g DW, respectively) (Figure 2). Extracts obtained by cold extraction from oven dried tissues exhibited significantly lower sucrose, glucose and fructose levels (5.2 ± 0.3 g/100g DW, 2.9 ± 0.1 g/100g DW and 4.6 ± 0.1 g/100g DW, respectively) compared to those obtained from freeze dried leaf samples (10.0 ± 0.3 g/100g DW, 8.0 ± 0.2 g/100 g DW and 9.4 ± 0.2 g/100g DW, respectively) (Figure 2).

### 2.2. Multivariate Analysis of the Data Obtained by Different Extraction Protocols

The obtained data was processed with Partial Least Squares–Discriminant Analysis (PLS-DA) to further clarify the relations between different extraction protocols. The PLS-DA model was employed due to the supervised nature of the learning algorithm. 

**Figure 3:**
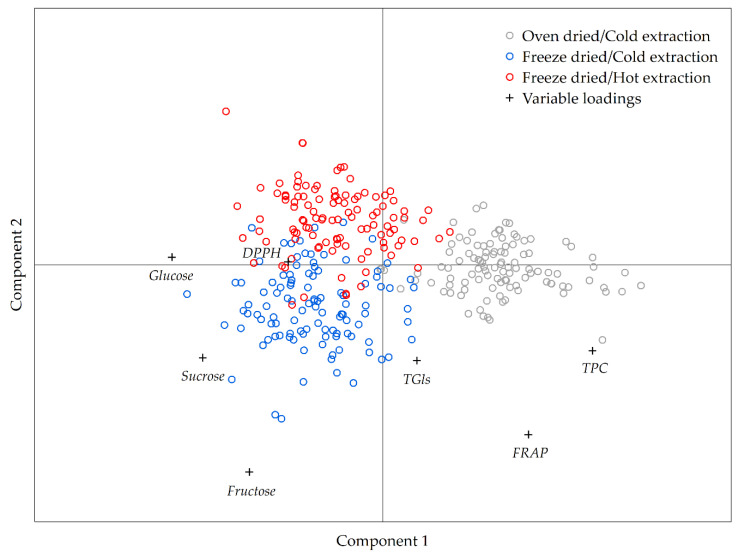
Partial Least Squares–Discriminant Analysis (PLS-DA) analysis of data obtained by different extraction protocols (N = 324); TGls—total glucosinolates; TPC—total phenolic content; DPPH—DPPH radical scavenging activity; FRAP—Ferric ion Reducing Antioxidant Power.

Overall, the extracts which included oven drying leaf tissue following cold methanol extraction were separated on the primary axis from the extracts obtained from freeze dried leaf tissue followed by cold or hot methanol extraction (Figure 3). The highest contribution to the sample placement on the primary axis had total phenolic content, DPPH radical scavenging activity, glucose, and sucrose content (Figure 3). On the second axis, the extracts, which were obtained from freeze dried leaf tissues following hot or cold methanol extraction, were separated according to fructose, total glucosinolates, and FRAP values (Figure 3).

The model showed that the largest difference between extraction protocols was in fructose content where the freeze drying and cold methanol combination yielded the highest amount of the compound. Total phenolic content was the second most important variable in the differentiation between the observed extraction protocols where the oven dried and cold methanol combination exhibited the highest yield of phenolic compounds. The third most important variable in differentiating among extraction protocols was glucose where similar amounts were extracted with either hot or cold methanol from freeze dried leaf tissue, whereas several-fold lower amounts were extracted from oven dried tissue with cold methanol. The next most important variable was FRAP where the highest values were observed in extracts obtained from oven dried tissues by cold methanol. Sucrose was the fifth most important variable where the oven dried leaf tissues had lower yields of the compound compared to extracts from freeze dried tissues. The sixth variable, according to the discriminating power, was DPPH radical scavenging, where again the extracts obtained from oven dried tissues exhibited lower antioxidant activity compared to extracts obtained from freeze dried leaf tissues. The variable with the lowest discriminating power according to the obtained model was total glucosinolates where cold methanol extraction from freeze dried Brassica tissue exhibited the highest yield compared to the other two extraction protocols. 

### 2.3. Correlations between Bioactive Compounds, Antioxidant Activity, and Sugar Content in Brassica Extracts

Statistically significant correlations (*p* ≥ 0.05) were observed between bioactive compounds content, antioxidant activity, as well as between sugar content (Table 1). Positive correlations were observed between total phenolic content, total glucosinolates, and FRAP values (Table 1). Negative correlations were observed between total phenolic content and sucrose, glucose, and fructose content, as well as total glucosinolates and glucose content (Table 1). DPPH scavenging activity correlated positively with sugar content but FRAP correlated negatively with sucrose and glucose content (Table 1). Positive correlations were observed between sucrose, glucose, and fructose content (Table 1). 

## 3. Discussion

Biological activity of kale containing phytochemicals are associated with antioxidant, anti-cancerogenic activity and protection of gastrointestinal and cardiovascular system [4,31,32,33]. Sikora et al. found that kale had much higher antioxidant activity than broccoli, Brussels sprouts, and cauliflower [34]. Šamec et al. on the other hand found that white cabbage and kale sprouts had significantly higher antioxidant capacity, polyphenol and glucosinolate content compared to arugula, broccoli, and Chinese cabbage sprouts [20]. Kale is also abundant in organic acids, including citric, malic, pyruvic, shikimic fumaric, and aconitic acids [35]. 

The freeze drying process is regarded as the best method for sample dehydration because it can prolong shelf life without compromising nutritional quality [36]. The major drawback of this method is limited sample quantity that can be processed simultaneously, long processing time (up to several days) and high energy consumption [37]. On the other hand, oven drying is readily accessible to most laboratories, sample quantity is seldom an issue and the drying time can be as low as one hour but rarely exceeds 24 h. 

In our study, freeze dried leaf tissues followed by cold extraction yielded significantly higher total glucosinolate content compared to either oven dried leaf tissue followed by cold extraction or freeze dried leaf tissue followed by hot extraction. The results are in accordance with Rutnakornpituk et al., who investigated the effect of freeze and oven drying on phenolic and glucosinolate content, as well as, antioxidant activity in five cruciferous vegetables where the authors concluded that freeze dried leaf tissues yielded higher glucosinolate content in all investigated species [38]. On the other hand, Tetteh et al. studied the effect of different various drying methods on post-harvest glucosinolate content in *Moringa oleifera* leaf tissues where no differences were observed between oven dried and freeze dried leaf tissue samples [39]. Doheny-Adams et al. investigated the effect of hot and cold methanol extraction on glucosinolate content in five Brassicaceae species [12]. The results from their study showed that the hot extraction step can be replaced with cold extraction with no losses in glucosinolate content, which is in line with the findings presented in our study [12]. 

Oven drying, compared to freeze drying followed by cold extraction yielded significantly higher total phenolic content and FRAP values and lower total glucosinolate content and DPPH scavenging activity in *B. oleracea* var. *acephala* leaf extracts. Similarly, Managa et al. showed that the majority of investigated phenolic compounds in Chinese cabbage significantly increased in content in oven dried compared to freeze dried samples [40]. Although the phenolic compound content in Chinese cabbage was higher, the antioxidant activity measured by FRAP, DPPH, and ABTS, was significantly lower in the oven dried compared to freeze dried samples [40]. On the other hand, Korus found that freeze dried kale had significantly higher total phenolic content and antioxidant activity compared to air dried kale, albeit at a lower temperature (55 °C) compared to this study (105 °C) [41]. Papoutsis et al. found higher total phenolic content and DPPH scavenging activity in hot air dried compared to freeze dried lemon [28]. Que et al. also found higher total phenolic content and antioxidant activity in hot air dried compared to freeze dried pumpkin flour [27]. Das et al. studied the antioxidant properties of freeze dried and oven dried wheatgrass where higher phenolic content but lower FRAP and DPPH radical scavenging activity was found in hot air dried compared to freeze dried samples [42]. Hot air drying also exhibited elevated total phenolic content and antioxidant activity compared to freeze drying in olive leaves as shown by Ahmad-Qasem et al. [43]. Hot air dried leaf tissue extracts tend to exhibit higher phenolic compounds content, as found in this study, but the link with antioxidant capacity is not clear. In our study we found FRAP having significant correlation with total glucosinolate content as well as total phenolic content, but no significant correlation with DPPH scavenging activity. Interestingly, we found at the same time the highest DPPH radical scavenging activity and lowest FRAP values in freeze dried tissues followed by hot extraction, while exactly the opposite was determined in hot air dried samples followed by cold extraction. The obtained results are contrary to previously published [42,44,45] data where FRAP and DPPH radical scavenging assays have high correlations, especially since it is known that both depend mainly on the electron transfer mechanism which measures the antioxidant’s reducing ability [46,47]. FRAP values correlated highly with total phenolic content and negatively with sucrose and fructose content while the data obtained by the DPPH radical scavenging assay correlated positively with the samples’ sugar content possibly indicating interferences with the meta-products of Maillard reactions occurring during the hot air drying process [48]. 

Hot air drying *B. oleracea* var. *acephala* leaf tissues followed by cold extraction induced significantly lower content of all investigated sugars compared to freeze drying followed by cold extraction. If freeze dried tissues were subjected to hot extraction sucrose and fructose levels were significantly lower compared to the cold extraction process but higher than oven dried leaf tissues except for fructose. Fante and Zapata Norena studied the quality of hot air dried and freeze dried garlic and found that inulin content decreased with hot air drying while glucose and fructose content increased compared to freeze dried garlic [49]. Iombor et al. studied changes in soursop flour composition as affected by oven and freeze drying and found 40% decrease in carbohydrate content in hot air dried samples [50]. Zhang et al. studied the changes in chestnut starch properties during different drying methods and found lower starch but higher reducing sugar content in oven dried compared to freeze dried samples [51]. Karaman et al. found lower fructose and glucose content in oven dried compared to freeze dried persimmon powders [52]. On the other hand, Gao et al. showed that there was no difference in sucrose, glucose and fructose content in oven and freeze dried jujube samples [53]. The observed decrease in sugar content in our study could be attributed to Maillard reactions occurring at elevated temperatures during hot air drying [54] or possibly the caramelization of sugars [55]. Michalska et al. determined that increased drying temperature of different plum cultivars resulted in decreased total sugar content while early and intermediate Maillard reaction products increased in content [48]. Similarly, Li et al. observed a decrease in reducing sugar content during hot air drying opposed to freeze drying of instant *Tremella fuciformis,* which the authors attributed to an intense Maillard reaction occurring between the carbonyl group of the reducing sugars and amino acids at elevated temperatures [56]. Previously published data on oligosaccharide and simple sugars extraction in various solvent mixtures and extraction temperatures showed that, with increased temperature, especially at the solvent boiling point, sugar content yields were higher [57]. We, on the other hand, observed lower yields of sucrose and fructose in freeze dried leaves hot extracts, compared to the cold extraction step while glucose levels remained unaffected. 

## 4. Materials and Methods 

### 4.1. Plant Material

**Figure 4:**
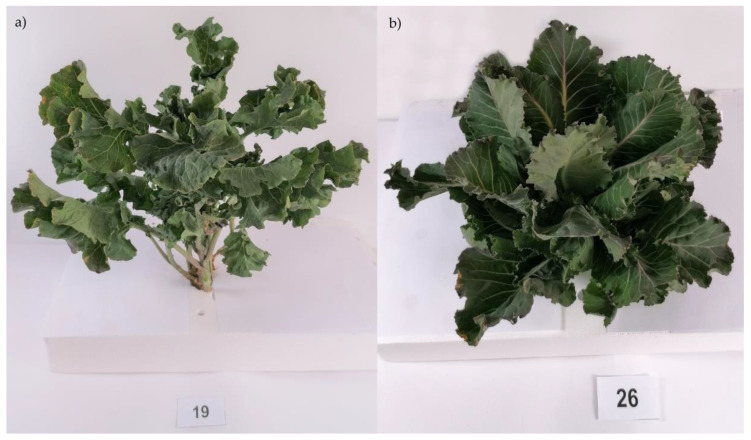
*Brassica oleracea* var. *acephala* accession (**a**) IPT399 and (**b**) IPT403.

Thirty-six *B. oleracea* var *acephala* ecotypes (accessions IPT379, IPT390, IPT386, IPT391, IPT381, IPT392, IPT393, IPT419, IPT394, IPT395, IPT383, IPT396, IPT384, IPT385, IPT397, IPT398, IPT420, IPT399, IPT400, IPT387, IPT401, IPT 402, IPT403, IPT404, IPT405, IPT406, IPT174, IPT202, IPT206, IPT407, IPT421, IPT408, IPT409, IPT410, IPT380, IPT411) used in this study were grown under the same agro-climatic conditions on the experimental farm of the Institute of Agriculture and Tourism, Poreč, Croatia (N 45°13′20.30″, E 13°36′6.49″) and are part of the National Program for the Conservation and Sustainable Use of Plant Genetic Resources for Food and Agriculture (accession IPT399 and IPT403 are shown in Figure 4). The leaves used in this study were harvested when the plants reached technological maturity. Harvested leaves were fully developed without any signs of physiological, pest or diseases injury. Fresh plant samples (three biological replicates per sample), immediately after harvesting, were either kept at −80 °C until the freeze drying process or placed in an oven (Memmert UF160, Schwabach, Germany) at 105 °C overnight. Frozen plant samples were placed in a freeze dryer (Labogene Coolsafe 95-15 Pro, Allerød, Denmark) and lyophilized over a period of 48 h. Lyophilized or oven-dried samples were ground to powder (0.2 mm) using an ultra-centrifugal mill (Retsch ZM200, Haan, Germany).

### 4.2. Hot Methanol Extraction 

Freeze dried plant material (30 mg) was preheated to 75 °C for 3 min in a heating/cooling dry block (Biosan CH100, Riga, Latvia) and 1.5 mL of preheated 70: 30 methanol:water (*v*/*v*) at 75 °C was added. The samples were incubated for 10 min at 75 °C and manually shaken every 2 min. Afterwards the samples were centrifuged at 15,000 G for 5 min (Domel Centric 350, Železniki, Slovenia) and the supernatant was filtered through a 0.22 µm nylon filter and transferred to a clean tube. The samples were stored at −80 °C until further analysis. 

### 4.3. Cold Methanol Extraction

Freeze dried or oven dried plant material (30 mg) was extracted with 1.5 mL of 80:20 methanol: water (*v*/*v*) at 20 °C over a period of 30 min in an ultrasonic bath (MRC 250H, Holon, Israel). The samples were centrifuged at 15,000 G for 5 min (Domel Centric 350, Železniki, Slovenia) and the supernatant was transferred to a clean tube. The samples were stored at −80 °C until further analysis.

### 4.4. Determination of Total Antioxidant Activity

Total antioxidant activity was evaluated using the FRAP assay [58] ant the DPPH radical scavenging activity assay [59]. Briefly, 100 µL of the sample was mixed with 200 µL of either freshly prepared FRAP reagent or 0.02M DPPH radical for the FRAP or DPPH assays, respectively. The antioxidant activity using the FRAP assay was evaluated after 10 min of reaction time at 25 °C by reading the absorbance at 593 nm while the DPPH radical scavenging ability was evaluated after 30 min of reaction time at 25 °C by reading the absorbance at 517 nm (Tecan Infinite 200 Pro M Nano+, Männedorf, Switzerland). FRAP values were calculated against a Fe^2+^ calibration curve (y = 0.0168x − 0.002; serial dilutions of Fe^2+^—20, 40, 80, 120, 160, 200, 250 µM; coefficient of determination, R^2^ = 0.9999, recovery: 101.8 ± 1.6 %) and expressed as µmol Fe^2+^/g DW. DPPH radical scavenging ability values were calculated against a standard curve of Trolox (y = −0.0128x + 0.0125; serial dilutions of Trolox—2, 5, 10, 25, 50, 75, 100 µM; coefficient of determination, R^2^ = 0.9995, recovery: 103.7 ± 1.2 %) and expressed as µmol TEQ/g DW, respectively.

### 4.5. Determination of Total Glucosinolates and Total Phenolic Content

Total glucosinolates were determined according to Ishida et al. [60] with some modifications. Briefly, 10 µL of plant extract was mixed with 300 µL of 2 mM Palladium (II) chloride and after 30 min of reaction time at 25 °C absorbance was read at 425 nm (Tecan Infinite 200 Pro M Nano+, Männedorf, Switzerland). The results were calculated against a standard curve of sinigrin (y = 3.8021x + 0.1682; serial dilutions of sinigrin—0.3, 0.6, 1.2, 1.8, 2.4, 3.0 mg/L; coefficient of determination, R^2^ = 0.9995, recovery: 99.7 ± 2.9%) and expressed as SEQ/g DW. Total phenolic content was determined according to Singleton and Rossi [61] with some modifications. The methanolic extracts (20 µL) were mixed with 140 µL of freshly prepared 0.2M Folin–Ciocalteu reagent. After 1 min, 140 µL of 6% solution of Calcium carbonate was added to the mixture. The absorbance was read at 750 nm (Tecan Infinite 200 Pro M Nano+, Männedorf, Switzerland) after 60 min of reaction time at 25 °C. The results were calculated against a standard curve of gallic acid (y = 3.7867x − 0.2144; serial dilutions of gallic acid—12.5, 25, 50, 75, 100, 150, 250 mg/L; coefficient of determination, R^2^ = 0.9999, recovery: 102.0 ± 2.9 %) and expressed as mg GAEQ/g DW.

### 4.6. Sugar Analysis by HPLC

The analysis of sucrose, fructose and sucrose content was carried out using a HPLC system consisting of a solvent delivery unit (Varian 210, Palo Alto, CA, USA), an autosampler (Varian 410, Palo Alto, CA, USA), column oven (Varian CM500, Palo Alto, CA, USA) and a refractive index detector (Varian 350, Palo Alto, CA, USA). Chromatographic separation was achieved by injecting 10 µL of the sample on a 300 × 8 mm, 9 µm particle size, calcium cation exchange column (Dr. Maisch ReproGel Ca, Ammerbuch, Germany) held at 80 °C using deionized water as the mobile phase (1 mL/min, isocratic elution). Retention times and peak areas of the investigated sugars were compared to analytical standards for identification and quantification, respectively. Linear calibration curves were obtained with serial dilutions of 0.25, 0.50, 1.00, 2.50, 5.00, 7.50, 10.00 g/L of sucrose (y = 2265.73x + 29.97, coefficient of determination, R^2^ = 0.9998, recovery: 99.9 ± 2.3 %), glucose (y = 2224.75x + 28.33, coefficient of determination, R^2^ = 0.9999, recovery: 99.8 ± 1.8 %) and fructose (y = 2233.53x + 47.37, coefficient of determination, R^2^ = 0.9998, recovery: 100.0 ± 0.6 %).

### 4.7. Statistical Analysis

To determine the effect of the investigated extraction protocols on total antioxidant activity, total glucosinolate, total phenolic and sugar content in *B. oleracea* var. *acephala* leaves the results were processed by analysis of variance (ANOVA) and reported as mean ± SE. Pearson’s correlations were calculated to evaluate the connection between bioactive compounds content, antioxidant activity, and sugar content. Further investigation on the impact of different extraction protocols on the studied compounds was carried out by employing PLS-DA as a supervised multivariate method. All statistical analyses were performed using Statistica 13.4.0.14. (Tibco Inc., Palo Alto, CA, USA). Significant differences were determined at *p* ≤ 0.05 and homogenous group means were compared by Tukey–Kramer Unequal N HSD test. 

## 5. Conclusions

The results from our study confirm the results published by Doheny-Adams et al. [12] that cold (ambient temperature) aqueous methanol can be used instead of boiling aqueous methanol with no adverse effects on total glucosinolate content. In addition to higher total glucosinolate content we observed an increase in antioxidant activity measured by FRAP, total phenolic content, sucrose content, and fructose content when the cold extraction step was applied on freeze dried *B. oleracea* var. *acephala* plant tissues while glucose levels remained unaffected. Our results also show that hot air drying, compared to freeze drying, followed by cold extraction has an adverse effect on antioxidant activity measured by DPPH radical scavenging, total glucosinolate content, as well as, on the content of all studied sugars in *B. oleracea* var. *acephala* leaves, indicating it would be inferior to freeze drying. On the other hand, if phenolics are the compounds of interest in *B. oleracea* var. *acephala* leaves, hot air drying may be a viable alternative to freeze drying.

## Figures and Tables

**Table 1:** Pearson’s correlations between bioactive compounds content, antioxidant activity and sugar content in B. oleracea var. acephala extracts (N = 324).

	TGls	TPC	DPPH	FRAP	Sucrose	Glucose	Fructose
TGls	1.00						
TPC	0.14 ^1^	1.00					
DPPH	0.06	−0.09	1.00				
FRAP	0.31	0.78	0.09	1.00			
Sucrose	−0.08	−0.44	0.27	−0.23	1.00		
Glucose	−0.13	−0.65	0.26	−0.41	0.47	1.00	
Fructose	−0.01	−0.23	0.12	0.09	0.55	0.43	1.00

^1^ Numbers highlighted in red are statistically significant (*p* ≤ 0.05); TGls—total glucosinolates; TPC—total phenolic content; DPPH—DPPH radical scavenging activity; FRAP—Ferric ion Reducing Antioxidant Power.
